# Association analysis of polymorphisms in *SLK*, *ARHGEF9*, *WWC2*, *GAB3,* and *FSHR* genes with reproductive traits in different sheep breeds

**DOI:** 10.3389/fgene.2024.1371872

**Published:** 2024-04-12

**Authors:** Meini Tao, Zhiqiang Li, Meng Liu, Haiyu Ma, Wujun Liu

**Affiliations:** ^1^ College of Animal Science, Xinjiang Agricultural University, Urumqi, China; ^2^ Adsen Biotechnology Co., Ltd., Urumchi, China

**Keywords:** sheep, fecundity, litter size, KASP, SNP, RT-qPCR

## Abstract

The aim was to investigate the relationship between polymorphisms of gene mutation loci and reproductive traits in local sheep breeds (Duolang Sheep) and introduced sheep breeds (Suffolk, Hu Sheep) in Xinjiang to provide new molecular markers for the selection and breeding of high fecundity sheep. The expression pattern of typing successful genes in sheep tissues was investigated by RT-qPCR technology, providing primary data for subsequent verification of gene function. The 26 mutation loci of *WWC2*, *ARHGEF9*, *SLK*, *GAB3*, and *FSHR* genes were typed using KASP. Association analyses were performed using SPSS 25.0, and the typing results showed that five genes with six loci, *WWC2* (g.14962207 C>T), *ARHGEF9* (g.48271079 C>A), *SLK* (g.27107842 T>C, g.27108855 G>A), *GAB3* (g.86134602 G>A), and *FSHR* (g.80789180 T>G) were successfully typed. The results of the association analyses showed that *WWC2* (g.14962207 C>T), *SLK* (g.27108855 G>A), *ARHGEF9* (g.48271079 C>A), and *FSHR* (g.80789180 T>G) caused significant or extremely significant effects on the litter size in Duolang, Suffolk and Hu Sheep populations. The expression distribution pattern of the five genes in 12 sheep reproduction-related tissues was examined by RT-qPCR. The results showed that the expression of the *SLK* gene in the uterus, the *FSHR* gene in the ovary, and the *ARHGEF9* gene in hypothalamic-pituitary-gonadal axis-related tissues were significantly higher than in the tissues of other parts of the sheep. *WWC2* and *GAB3* genes were highly expressed both in reproductive organs and visceral tissues. In summary, the *WWC2* (g.14962207 C>T), *SLK* (g.27108855 G>A), *ARHGEF9* (g.48271079 C>A), and *FSHR* (g.80789180 T>G) loci can be used as potential molecular markers for detecting differences in reproductive performance in sheep. Due to variations in typing results, the *SLK* (g.27107842 T>C) and *GAB3* (g.86134602 G>A) loci need further validation.

## 1 Introduction

China, as the country with the most significant amount of sheep rearing, slaughtering, and mutton output, meat sheep farming has gradually transitioned from traditional farmer farming to large-scale and industrialized agriculture, but because most of the meat sheep breeds farmed in China are mainly single lamb, with low fecundity, which increases the cost of production and the expansion of the farming scale is slow, which seriously restricts the rapid development of the meat sheep industry in China (Wang et al., 2018). As one of its most important economic traits, the reproductive performance of sheep directly determines the cost and efficiency of sheep farming production. Increasing the litter size of sheep parity is the most direct way to improve production efficiency, which is of great economic significance. Litter size is a quantitative trait that is controlled by multiple genes. Because of the complexity of the factors affecting the lambing trait and the low heritability of this trait, the genetic mechanism has not been thoroughly investigated, and the complex genetic mechanism of the lambing trait also makes the cycle of traditional selection methods to improve it long (Tao et al., 2020). Marker-assisted selection has the advantages of high efficiency, accuracy, and maneuverability, which can solve inefficiency problems and improve breeding efficiency.

The *WWC2* gene is a member of the WWC family of proteins. The WWC proteins and cytoplasmic core kinase cascade work together to regulate Hippo signaling, a pathway that is critical for early embryonic development (Nishioka et al., 2009; Wicklow et al., 2014; Posfai et al., 2017). Impaired transduction inhibits apoptosis and enhances cell proliferation (Meng et al., 2016; Bae and Luo, 2018; Ma et al., 2019). The *SLK* gene is a member of the Ste20 family of non-receptor serine/threonine protein kinases involved in cell proliferation, migration, and terminal differentiation processes (Davis, 1993; Fanger et al., 1997). *SLK* genes are classified as GCK-related kinases (Kuramochi et al., 1997) and are commonly expressed in adult tissues and cell lines and developing mouse embryos (Sabourin and Rudnicki, 1999; Zhang et al., 2002). Recent studies have identified an association between the *ARHGEF9* gene and seasonal estrus-related genes such as *DIO2* and *DIO3* in the thyroid (TH) axis (Fan et al., 2022). The *ARHGEF9* gene encodes a Rho-like GTPase involved in energy metabolism (Ibaraki et al., 2018), which may be a key candidate gene affecting seasonal estrus in sheep. *GAB3* gene deficient mice exhibit impaired uterine NK cell dilation associated with abnormal spiral artery remodeling and increased basal molting trophoblast invasion, leading to stillbirth, retained placenta, maternal hemorrhage, and undelivered placenta of full-term fetuses, which limits trophoblast invasion during pregnancy (Sliz et al., 2019). Follicle-stimulating hormone (FSH) is secreted by the anterior pituitary gland and plays a crucial role in reproduction (Gharib et al., 1990), it is essential for follicular growth, development, differentiation, and triggering follicular maturation and ovulation.

Molecular marker screening is an integral part of molecular breeding. SNP markers are the third generation of molecular markers and are currently dominant. KASP (Kompetitive allele specific polymerase chain reaction) technology is now an essential tool for genotyping. Originally named competitive allele specific PCR (KASP) by its developers, the method is a fluorescence detection-based variant of AS-PCR applicable to amplification results (Alvarez-Fernandez et al., 2021). KASP is primarily used in a wide range of genomic DNA samples for SNPs and locus-specific InDels for precise double allele determination for genotyping. Compared with other SNP detection methods, KASP technology has higher throughput, faster speed, lower cost, and more accurate results (Semagn et al., 2014; Landoulsi et al., 2017). Wang et al. (2022) used KASP genotyping to analyze the correlation between *PPP2R5C* and *SLC39A5* polymorphisms and litter size in black goats on Yunshang. Kusza et al. (2018) studied 48 SNPs affecting milk production traits in goats using KASP technology. Talebi et al. (2018) analyzed the correlation between single nucleotide polymorphisms in significant prolificacy genes and litter size in Mehraban sheep. These studies validated the reliability of KASP results.

In this experiment, Duolang, Hu, and Suffolk Sheep were used as research subjects, and five reproductive performance-related genes (*WWC2*, *SLK*, *ARHGEF9*, *GAB3*, and *FSHR*) were obtained by using the results of the team’s previous litter size trait GWAS analysis, based on the existing *WWC2*, *SLK*, *ARHGEF9*, *GAB3*, and *FSHR* in Ensembl and UCSC databases, we used KASP technology to type the mutation loci, to investigate the relationship between the polymorphisms of *WWC2*, *SLK*, *ARHGEF9*, *GAB3,* and *FSHR* loci and the reproductive performance in different sheep populations, and to screen the key loci affecting the reproductive performance of sheep. At the same time, the expression and distribution patterns of *WWC2*, *SLK*, *ARHGEF9*, *GAB3*, and *FSHR* genes in 12 different reproduction-related tissues of sheep breeds in Xinjiang were investigated, which provided the preliminary primary data for further validation of gene functions. Due to different living environments, the reproductive performance of sheep varies from breed, and there are also differences within the breed. The main genes that cause differences in sheep litter size are also different. Therefore, the objectives of this study were to screen for new candidate genes and molecular markers that are most likely to affect sheep reproductive traits.

## 2 Materials and methods

### 2.1 Sample collection

The study was conducted on 300 Duolang sheep (lambing one or more lambs in a single litter), 167 Hu sheep (lambing one or more lambs in a single litter), and 100 Suffolk (lambing one or more lambs in a single litter). The Duolang sheep were from the sheep farms of Xinjiang Maigaiti Daolang Sunshine Agricultural and Animal Husbandry Science and Technology Co., Ltd. The Hu sheep were from the sheep farms of Hami Jiankun Farming Co. The Suffolk sheep were from the sheep farms of Manasi Rifa. The blood samples were collected from three breeds of sheep, and one blood sample was collected from the jugular vein of each sheep, put into vacuum blood collection tubes containing anticoagulant factor (EDTA K2), and stored at −20°C storage backup.

Taking sheep in the Xinjiang region as the test object, three healthy ewes with the same parity were selected, requiring the same feeding environment, basically the same or similar body weight, and prohibited from eating for 24 h before slaughtering and determination. In this test, the heart, liver, spleen, lungs, kidneys, uterine horns, uterus, ovary, hypothalamus, pituitary gland, brain, and cerebellum were taken as tissue samples quickly after slaughtering the sheep. Three copies were taken from each sample and then put into the liquid nitrogen immediately afterward. The subsequent determination and analysis could be done by extracting the RNA from the tissue samples.

### 2.2 Phenotype data collection

According to the sheep’s lambing pattern, contact the corresponding sheep farms and record the lambing data of sheep in different parity (Supplementary Table S1).

### 2.3 Blood DNA, tissue RNA extraction and detection

DNA was extracted from sheep blood samples (300 Duolang, 167 Hu, and 100 Suffolk sheep) using the blood/cell/tissue genomic DNA extraction kit (TIANGEN, Beijing, China), and the DNA quality was detected by 1.5% agarose gel electrophoresis. Tissue RNA was extracted using the Biomarker Cell/Tissue Total RNA Isolation Kit (Biomarker Technologies, Beijing, China). Multiskan GO (Thermo Fisher Scientific, Wilmington, MA, United States) determined RNA’s purity, and the RNA quality was detected by 1.5% agarose gel electrophoresis. The cDNA was synthesized using the TIANGEN Fastking cDNA First Strand Synthesis Kit (TIANGEN, Beijing, China).

### 2.4 KASP typing test

The SNP information of five genes (*WWC2*, *SLK*, *ARHGEF9*, *GAB3*, *FSHR*) was searched using Ensembl (https://useast.ensembl.org/index.html) and UCSC website (https://genome-asia.ucsc.edu/index.html). The 26 missense mutation sites in the five genes were selected for KASP typing, and the primers were designed using the DNAMAN 8.0 software. Biomarker Technologies Co., Ltd. (Beijing, China) synthesized the primers. Mutation sites and primers information (Table 1).

**TABLE 1:** Information of mutation sites and primers.

Number	Gene	SNP	Position	Alleles	Primer sequence
1	*WWC2*	rs602708321	26:14,923,661	G/A	F1:GAAGGTCGGAGTCAACGGATTTACAAGGAGAAGTCCAGCTCTCA
					F2:GAAGGTGACCAAGTTCATGCTCAAGGAGAAGTCCAGCTCTCG
					R:CTCAGGTCTGAACAGCCACG
2		rs602191706	26:14,937,763	A/G	F1:GAAGGTCGGAGTCAACGGATTGAGCAATTCCTGCTTCATCC
					F2:GAAGGTGACCAAGTTCATGCTATAGAGCAATTCCTGCTTCATCT
					R:AAAAAGCTGAAGCGAGAACTCT
3		rs424372214	26:14,944,442	G/T	F1:GAAGGTCGGAGTCAACGGATTCTGGTTTGTGAGCCCGC
					F2:GAAGGTGACCAAGTTCATGCTGCTGGTTTGTGAGCCCGA
					R:TCCCTCTCCAGACAGACCCT
4		rs400706256	26:14,962,207	C/T	F1:GAAGGTCGGAGTCAACGGATTTGCGGTTCAGATTAAAATCCA
					F2:GAAGGTGACCAAGTTCATGCTGCGGTTCAGATTAAAATCCG
					R:CGTGGGTATCGGCGTCTAT
5		rs408331918	26:14,960,313	C/T	F1:GAAGGTCGGAGTCAACGGATTAGGCCGAGGACGTGCC
					F2:GAAGGTGACCAAGTTCATGCTGAGGCCGAGGACGTGCT
					R:TGATCGCAGCGTCCTCCT
6	*ARHGEF9*	rs429786009	X:48,271,079	C/A	F1:GAAGGTCGGAGTCAACGGATTGACTTCTCCAGACCTACCTCCA
					F2:GAAGGTGACCAAGTTCATGCTGACTTCTCCAGACCTACCTCCC
					R:AGGCCACCCTGAGGTAGAAG
7		rs595496096	X:48,270,962	C/T	F1:GAAGGTCGGAGTCAACGGATTACTGGAGGCCCCTTTTCTAT
					F2:GAAGGTGACCAAGTTCATGCTACTGGAGGCCCCTTTTCTAC
					R:CAAGAAGAAAATAAAAGTCAACAAGAT
8		rs161682190	X:48,270,275	C/T	F1:GAAGGTCGGAGTCAACGGATTACCACTGATGAAGTAGGTCTTGC
					F2:GAAGGTGACCAAGTTCATGCTCACCACTGATGAAGTAGGTCTTGT
					R:AAGTGGCGTGCATTATCCC
9		rs1089770370	X:48,270,113	G/A	F1:GAAGGTCGGAGTCAACGGATTCCCTCAATGCTCATTTCAAATA
					F2:GAAGGTGACCAAGTTCATGCTCCCTCAATGCTCATTTCAAATG
					R:CCAGCACTGTCACCAAAAGG
10		rs1090368365	X:47,951,753	C/T	F1:GAAGGTCGGAGTCAACGGATTAAGTCATCATCTCTGCCATCTTT
					F2:GAAGGTGACCAAGTTCATGCTAAGTCATCATCTCTGCCATCTTC
					R:TATGGATAAATATGAGGTAGTGGACA
11		rs595001753	X:48,270,705	A/T	F1:GAAGGTCGGAGTCAACGGATTAAAGCCTTTTCGTTATTCCTACA
					F2:GAAGGTGACCAAGTTCATGCTCAAAGCCTTTTCGTTATTCCTACT
					R:CTCCAGGCCCTCAGTGTGT
12		rs427586477	X:48,269,718	C/T	F1:GAAGGTCGGAGTCAACGGATTCCCCAGGATTCTGACTTGGATA
					F2:GAAGGTGACCAAGTTCATGCTCCCAGGATTCTGACTTGGATG
					R:TGGCTTTCCTCTCTGCACTTG
13	*SLK*	rs402599671	22:27,107,842	T/C	F1:GAAGGTCGGAGTCAACGGATTAAATTTATCTTCAGAAGTATTACGTTCTA
					F2:GAAGGTGACCAAGTTCATGCTAATTTATCTTCAGAAGTATTACGTTCTG
					R:GTATTTTGGAATCTGTCTCGGAA
14		rs413475281	22:27,108,855	G/A	F1:GAAGGTCGGAGTCAACGGATTGCAGAACCTCAAGTTATTGCCA
					F2:GAAGGTGACCAAGTTCATGCTCAGAACCTCAAGTTATTGCCG
					R:TGGGTATTGGAACAGGCTGA
15		rs1089495174	22:27,113,129	C/T	F1:GAAGGTCGGAGTCAACGGATTTGCGAGATGAAGCCAAGC
					F2:GAAGGTGACCAAGTTCATGCTCTTGCGAGATGAAGCCAAGT
					R:GCTCTTTTTCTTGGTCACCTTTA
16		rs1086178068	22:27,115,681	C/T	F1:GAAGGTCGGAGTCAACGGATTGCAAGCTCCTCTTTCCTGCA
					F2:GAAGGTGACCAAGTTCATGCTCAAGCTCCTCTTTCCTGCG
					R:AGTGGAGAGAGCACCCAAAGA
17		rs592101884	22:27,130,196	G/T	F1:GAAGGTCGGAGTCAACGGATTAAAAGACACTGGAAGAAGAGTTTG
					F2:GAAGGTGACCAAGTTCATGCTAAAAAGACACTGGAAGAAGAGTTTT
					R:ACACTTCCTGTTCCTGTAGTTTCC
18	*GAB3*	rs414255535	X:86,134,726	A/C	F1:GAAGGTCGGAGTCAACGGATTCATCATGTCTAGTTCTTTTACCCC
					F2:GAAGGTGACCAAGTTCATGCTACATCATGTCTAGTTCTTTTACCCA
					R:CATCCAGGTAGATCAAAACCAAG
19		rs591630593	X:86,134,602	G/A	F1:GAAGGTCGGAGTCAACGGATTGCACCACAGTGCCTTCCA
					F2:GAAGGTGACCAAGTTCATGCTGCACCACAGTGCCTTCCG
					R:AGCAGTTCCTGTGCAGAGGG
20		rs429242422	X:86,097,039	C/T	F1:GAAGGTCGGAGTCAACGGATTTCCACCAGCACTGAGGACA
					F2:GAAGGTGACCAAGTTCATGCTCCACCAGCACTGAGGACG
					R:GGGGACTCATGGGCACAT
21		rs1088451252	X:86,077,337	C/T	F1:GAAGGTCGGAGTCAACGGATTGACATTGGTTTCTAGGAGAAGCA
					F2:GAAGGTGACCAAGTTCATGCTACATTGGTTTCTAGGAGAAGCG
					R:CCACTGCTCAAGGAGTTGGTAG
22	*FSHR*	rs1093370251	3:80,789,131	G/A	F1:GAAGGTCGGAGTCAACGGATTAGTGGTAAGTTGACTTGGCCC
					F2:GAAGGTGACCAAGTTCATGCTTAAGTGGTAAGTTGACTTGGCCT
					R:GCCTAGCTATGGCTTAGAAAATCT
23		rs422112895	3:80,789,180	T/G	F1:GAAGGTCGGAGTCAACGGATTGCTTCCCAGTCTGGAAAAATTG
					F2:GAAGGTGACCAAGTTCATGCTGCTTCCCAGTCTGGAAAAATTT
					R:AGGCTGGCCTCCACGAGT
24		rs1092181549	3:80,794,798	T/A	F1:GAAGGTCGGAGTCAACGGATTGACATCAACTGAGGCTATGAGCA
					F2:GAAGGTGACCAAGTTCATGCTGACATCAACTGAGGCTATGAGCT
					R:CCGATCTCTGCATTGGAATCTA
25		rs1093458544	3:80,794,420	C/T	F1:GAAGGTCGGAGTCAACGGATTTTTTCTAGCTCTGACCTTCATCC
					F2:GAAGGTGACCAAGTTCATGCTATTTTTCTAGCTCTGACCTTCATCT
					R:CTTGCCTTAAAATAGATTTGTTGC
26		rs1094538748	3:80,795,002	G/A	F1:GAAGGTCGGAGTCAACGGATTAATGCAAAGTGCAGCTCCA
					F2:GAAGGTGACCAAGTTCATGCTATGCAAAGTGCAGCTCCG
					R:AGACCCAGCCCACCAGC

### 2.5 RT-qPCR

The genes that were successfully typed by KASP (*WWC2*, *ARHGEF9*, *SLK*, *GAB3*, and *FSHR*) were selected for RT-qPCR test, and 12 different reproduction-related tissues of sheep in Xinjiang region (heart, liver, spleen, lungs, kidneys, uterine horns, uterus, ovary, hypothalamus, pituitary gland, brain, cerebellum) were used as templates for the cDNA. The *β-actin* gene was used as an internal reference, and three replicates were performed for each sample. The sequences of the target genes were found using the NCBI online website (https://www.ncbi.nlm.nih.gov/) and compared in the UCSC Sheep Genome Database (https://genome-asia.ucsc.edu/cgi-bin/hgGateway) to identify the exon locations. The NCBI online website (https://www.ncbi.nlm.nih.gov/tools/primer-blast/index.cgi?LINK_LOC=BlastHome) was utilized to design six genes (*WWC2*, *ARHGEF9*, *SLK*, *GAB3*, and *FSHR*) for the Primers were synthesized by Xinjiang Youkang Biotechnology Co., Ltd. (Urumqi, China). Gene sequence information (Table 2).

**TABLE 2:** Gene sequence information.

Gene	Sequence(5′-3′)	Primer sequence	Length/bp
*WWC2*	XM_027962647.2	F:AGGCCTCGGATGAAACTGTG	116
		R:TGCGTCCTCATTCATCTCGG	
*ARHGEF9*	XM_015104868.3	F:TTTAAGGCTGGCGACGTCAT	102
		R:CACAAAGCTGGCAGGAAACC	
*SLK*	XM_004020168.5	F:ATCGCTTGCGAGATGAAGCC	170
		R:TTGTGCAAGCTCCTCTTTCCT	
*GAB3*	XM_042241607.1	F:TCGCACATTCTATCTGGTGGC	114
		R:TGGCCTTCGACATTTGTGCTG	
*FSHR*	XM_027965759.2	F:CAAGGTGACAGAGATGCCC	179
		R:GCAGGTTGGAGAACACATTTG	
*ACTB (β-actin)*	NM_001009784.3	F:ATCGTCCGTGACATCAAGG	178
		R:GGAAGGAAGGCTGGAAGAG	

### 2.6 Data analysis

(1) After successful typing of loci using the KASP typing technique, the collected production data and experimental data were statistically processed using an Excel sheet [Genotype frequency, Allele frequency, Expected heterozygosity (He), Effective allele numbers (Ne), Observed homozygosity (Ho), Polymorphic information content (PIC)]. Hardy-Weinberg equilibrium (HWE) was analyzed using the PowerMarker V3.25 chi-square test method (Liu and Muse, 2005). The relationship between genetic polymorphisms and litter size of Duolang, Suffolk, and Hu sheep was statistically analyzed using one-way ANOVA and *t*-test in SPSS 25.0, and the litter size was expressed as “mean ± standard deviation.”
(2) Relative quantitative analysis was performed using the 2^−ΔΔCt^ method (Livak and Schmittgen, 2001) with Excel software, and the quantitative data were visualized by GraphPad Prism 8.0 software.

## 3 Result

### 3.1 KASP typing results of 6 loci in 3 populations

**FIGURE 1:**
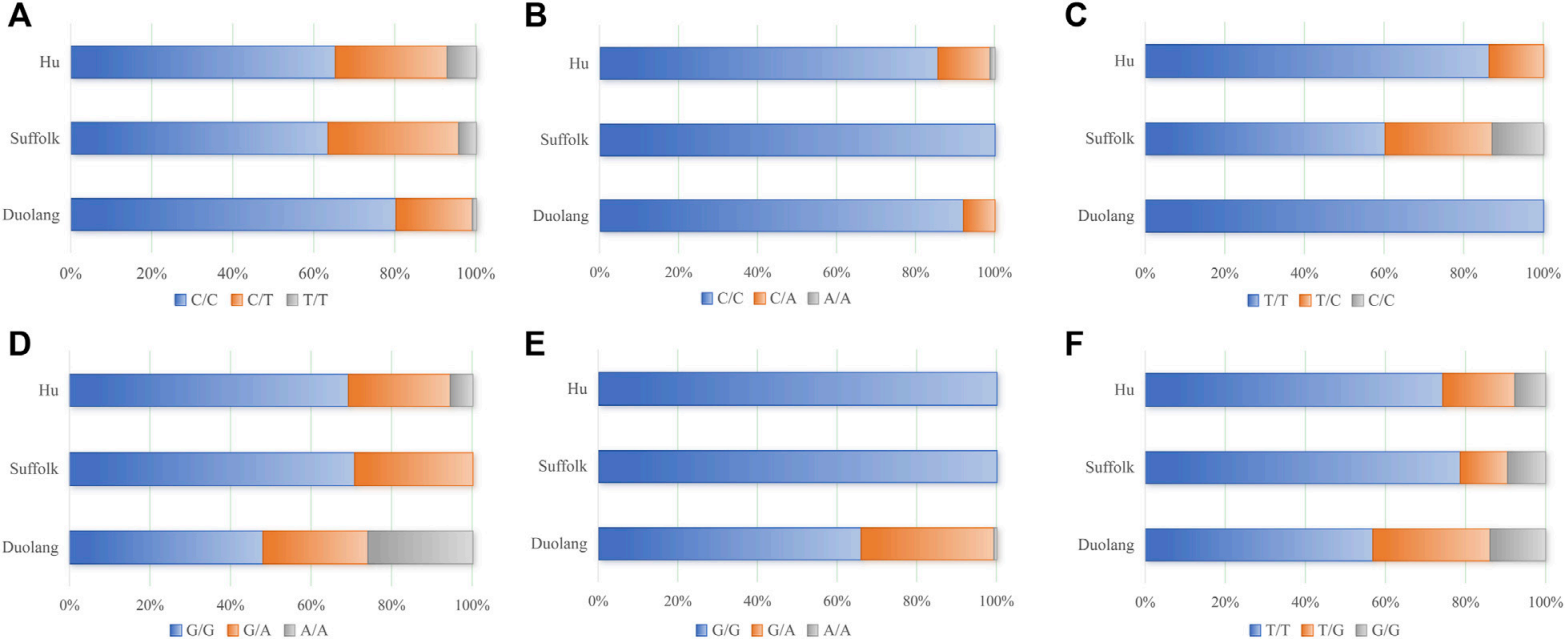
**(A)** Genotyping results of *WWC2* gene (g.14962207 C>T), **(B)** Genotyping results of *ARHGEF9* gene (g.48271079 C>A), **(C)** Genotyping results of *SLK* gene (g.27107842 T>C), **(D)** Genotyping results of *SLK* gene (g.27108855 G>A), **(E)** Genotyping results of *GAB3* gene (g.86134602 G>A), **(F)** Genotyping results of *FSHR* gene (g.80789180 T>G).

KASP typing was performed on 26 missense mutation sites of five genes (*WWC2*, *ARHGEF9*, *SLK*, *GAB3*, and *FSHR*). Finally, six loci of 5 genes (*WWC2*, *ARHGEF9*, *SLK*, *GAB3*, and *FSHR*) were successfully typed (Figure 1).

### 3.2 Genetic diversity analysis of *WWC2*, *SLK*, *ARHGEF9*, *GAB3*, and *FSHR* genes

#### 3.2.1 Genetic diversity analysis of the *WWC2* locus in three sheep breeds

The *WWC2* gene g.14962207 C>T locus was polymorphic in all three sheep populations, and the wild-type genotype frequency was higher than the heterozygous and mutant genotype frequencies in all three populations. The *WWC2* gene was in Hardy-Weinberg equilibrium (*p* > 0.05) in the Duolang and Suffolk Sheep populations and unbalanced (*p* < 0.05) in the Hu Sheep populations. The g.14962207 C>T locus of the *WWC2* gene showed low polymorphism (PIC<0.25) in the Duolang sheep populations and moderate polymorphism (0.25<PIC<0.5) in the Suffolk and Hu sheep populations (Table 3).

**TABLE 3:** Genetic diversity analysis of *WWC2* gene locus in three sheep breeds.

Locus	Sheep breeds	Genotype frequency			*p*-Value	Gene frequency		Ho	He	Ne	PIC
		CC	CT	TT		C	T				
g.14962207	Duolang	0.806	0.188	0.010	0.926	0.899	0.104	0.820	0.180	1.220	0.163
	Suffolk	0.634	0.323	0.043	0.940	0.796	0.204	0.675	0.325	1.482	0.272
	Hu	0.653	0.275	0.072	0.029	0.790	0.210	0.669	0.331	1.495	0.276

#### 3.2.2 Genetic diversity analysis of different loci of the *SLK* gene in three sheep breeds

The *SLK* gene g.27107842 T>C locus was polymorphic in Suffolk and Hu Sheep populations, and the *SLK* gene g.27108855 G>A locus was polymorphic in all three sheep populations. The wild-type genotype frequency was higher than the heterozygous and mutant genotype frequencies. The *SLK* gene g.27107842 T>C and g.27108855 G>A loci were in Hardy-Weinberg equilibrium (*p* > 0.05) in Suffolk and Hu Sheep populations and were unbalanced (*p* < 0.05) in Duolang Sheep populations. The g.27107842 T>C locus of the *SLK* gene in the Hu sheep populations showed low polymorphism (PIC<0.25) and moderate polymorphism (0.25<PIC<0.5) in the Suffolk sheep populations. *SLK* gene g.27108855 G>A locus showed moderate polymorphism (0.25<PIC<0.5) in both Duolang and Hu sheep populations and low polymorphism (PIC<0.25) in the Suffolk sheep populations (Table 4).

**TABLE 4:** Genetic diversity analysis of *SLK* gene locus in three sheep breeds.

Locus	Sheep breeds	Genotype frequency			*p*-Value	Gene frequency		Ho	He	Ne	PIC
		TT	TC	CC		T	C				
g.27107842	Duolang	1	—	—	—	1	—	1	—	1	—
	Suffolk	0.602	0.269	0.129	0.323	0.737	0.263	0.612	0.388	1.634	0.313
	Hu	0.862	0.138	—	0.339	0.931	0.069	0.872	0.128	1.147	0.120
		GG	GA	AA		G	A				
g.27108855	Duolang	0.479	0.260	0.260	0.041	0.609	0.391	0.524	0.476	1.909	0.363
	Suffolk	0.707	0.293	—	0.099	0.853	0.147	0.750	0.250	1.334	0.219
	Hu	0.693	0.253	0.054	0.061	0.819	0.181	0.704	0.296	1.421	0.252

#### 3.2.3 Genetic diversity analysis of the *ARHGEF9* locus in three sheep breeds

The g.48271079 C>A locus of the *ARHGEF9* gene was polymorphic in Duolang and Hu Sheep populations and the frequencies of the wild-type genotypes were higher than the frequencies of heterozygous and mutant-pure genotypes. The *ARHGEF9* gene was in Hardy-Weinberg equilibrium in the Duolang and Hu Sheep populations (*p* > 0.05). The g.48271079 C>A locus showed low polymorphism (PIC<0.25) in both the Duolang and Hu Sheep populations (Table 5).

**TABLE 5:** Genetic diversity analysis of *ARHGEF9* gene locus in three sheep breeds.

Locus	Sheep breeds	Genotype frequency			*p*-Value	Gene frequency		Ho	He	Ne	PIC
		CC	CA	AA		C	A				
g.48271079	Duolang	0.920	0.080	—	0.480	0.960	0.040	0.923	0.077	1.083	0.074
	Suffolk	1	—	—	—	1	—	1	—	1	—
	Hu	0.856	0.132	0.012	0.287	0.922	0.078	0.856	0.144	1.168	0.133

#### 3.2.4 Genetic diversity analysis of the *GAB3* locus in three sheep breeds

The *GAB3* gene g.86134602 G>A locus was polymorphic in the Duolang Sheep population, and the frequency of the wild-type genotype was higher than the heterozygous and mutant genotypes. The *GAB3* gene g.86134602 G>A locus was not in Hardy-Weinberg equilibrium (*p* < 0.05) in the Duolang Sheep populations. The *GAB3* gene g.86134602 G>A locus showed low polymorphism (PIC<0.25) in Duolang Sheep populations (Table 6).

**TABLE 6:** Genetic diversity analysis of *GAB3* gene locus in three sheep breeds.

Locus	Sheep breeds	Genotype frequency			*p*-Value	Gene frequency		Ho	He	Ne	PIC
		GG	GA	AA		G	A				
g.86134602	Duolang	0.660	0.333	0.007	0.036	0.826	0.174	0.713	0.287	1.402	0.246
	Suffolk	1	—	—	—	1	—	1	—	1	—
	Hu	1	—	—	—	1	—	1	—	1	—

#### 3.2.5 Genetic diversity analysis of different loci of the *FSHR* gene in three sheep breeds

The *FSHR* gene g.80789180 T>G locus was polymorphic in all three populations, and the wild-type genotype frequency was higher than the heterozygous and mutant genotype frequency in all three populations. The g.80789180 T>G locus of the *FSHR* gene was in Hardy-Weinberg equilibrium in all three populations (*p* > 0.05). The g.80789180 T>G locus showed low polymorphism (PIC<0.25) in the Suffolk and Hu Sheep populations and moderate polymorphism (0.25<PIC<0.5) in the Duolang Sheep populations (Table 7).

**TABLE 7:** Genetic diversity analysis of *FSHR* gene locus in three sheep breeds.

Locus	Sheep breeds	Genotype frequency			*p*-Value	Gene frequency		Ho	He	Ne	PIC
		TT	TG	GG		T	G				
g.80789180	Duolang	0.566	0.295	0.139	0.933	0.714	0.286	0.591	0.409	1.691	0.325
	Suffolk	0.785	0.118	0.097	0.935	0.844	0.156	0.737	0.263	1.357	0.229
	Hu	0.743	0.180	0.078	0.953	0.832	0.168	0.721	0.279	1.387	0.240

### 3.3 Association analysis between polymorphic loci of genes and litter size in three breeds of sheep

Polymorphisms in the *WWC2* gene g.14962207 C>T locus were significantly associated with the litter size in Duolang Sheep, the litter size of the wild-type CC genotype individuals was significantly higher than the heterozygous CT genotype individuals (*p* < 0.05). The litter size of the wild-type CC genotype individuals was significantly higher than the heterozygous CT genotype individuals (*p* < 0.01) and significantly higher than the mutant pure TT genotype individuals (*p* < 0.05) in the Suffolk Sheep populations. The *SLK* gene g.27107842 T>C locus in Suffolk Sheep populations, the litter size of mutant-pure CC genotype individuals was significantly higher than the wild-type TT genotype individuals and heterozygous TC genotype individuals (*p* < 0.01). The litter size of wild-type TT genotype individuals was significantly higher than the heterozygous TC genotype individuals in the Hu Sheep populations (*p* < 0.01). The *SLK* gene g.27108855 G>A locus in Duolang Sheep populations, the litter size of mutant-pure AA genotype individuals was significantly higher than the wild-type GG genotype individuals and heterozygous GA genotype individuals (*p* < 0.05), and the litter size of wild-type GG genotype individuals was significantly higher than the heterozygous GA genotype individuals in the Suffolk and Hu Sheep populations (*p* < 0.01). The *ARHGEF9* gene g.48271079 C>A locus in Hu Sheep populations, the litter size of heterozygous CA genotype individuals was significantly higher than the wild-type CC genotype individuals (*p* < 0.01). The *GAB3* gene g.86134602 G>A locus in Duolang Sheep populations, the litter size of mutant-pure AA genotype individuals and heterozygous GA genotype individuals was significantly higher than the wild-type GG genotype individuals (*p* < 0.01). The *FSHR* gene g.80789180 T>G locus in Duolang and Suffolk Sheep populations, the litter size of mutant-pure GG genotype individuals was significantly higher than the wild-type TT genotype individuals (*p* < 0.01) (Table 8).

**TABLE 8:** Association analysis between different genotypes and average litter size, different uppercase letters of the shoulder mark are highly significant (*p* < 0.01), different lowercase shoulder tags are significantly different (*p* < 0.05), and the same letter is not significantly different (*p* > 0.05), the same as below.

Gene	Locus	Sheep breeds	Genotype		
			CC	CT	TT
*WWC2*	g.14962207	Duolang	1.680 ± 0.468^a^	1.500 ± 0.505^b^	1.333 ± 0.577^ab^
		Suffolk	1.779 ± 0.418^Aa^	1.133 ± 0.346^Bb^	1.250 ± 0.500^ABb^
		Hu	1.596 ± 0.4928	1.500 ± 0.5055	1.500 ± 0.522
			CC	CA	AA
*ARHGEF9*	g.48271079	Duolang	1.498 ± 0.501	1.522 ± 0.511	—
		Suffolk	1.215 ± 0.413	—	—
		Hu	1.490 ± 0.502^B^	1.955 ± 0.213^A^	1.500 ± 0.707^AB^
			TT	TC	CC
*SLK*	g.27107842	Duolang	1.500 ± 0.5008	—	—
		Suffolk	1.286 ± 0.456^B^	1.120 ± 0.332^B^	1.917 ± 0.289^A^
		Hu	1.646 ± 0.480^A^	1.043 ± 0.209^B^	—
			GG	GA	AA
*SLK*	g.27108855	Duolang	1.493 ± 0.5017^b^	1.493 ± 0.503^b^	1.667 ± 0.475^a^
		Suffolk	1.261 ± 0.443^A^	1.111 ± 0.320^B^	—
		Hu	1.670 ± 0.472^A^	1.405 ± 0.497^B^	1.667 ± 0.500^AB^
			GG	GA	AA
*GAB3*	g.86134602	Duolang	1.516 ± 0.501^B^	1.677 ± 0.470^A^	1.500 ± 0.707^A^
		Suffolk	1.215 ± 0.413	—	—
		Hu	1.563 ± 0.498	—	—
			TT	TG	GG
*FSHR*	g.80789180	Duolang	1.463 ± 0.500^Bb^	1.600 ± 0.493^ABa^	1.750 ± 0.439^Aa^
		Suffolk	1.176 ± 0.383^B^	1.364 ± 0.505^AB^	1.667 ± 0.500^A^
		Hu	1.637 ± 0.483	1.767 ± 0.430	1.538 ± 0.519

### 3.4 Distribution pattern of differential gene expression in tissues

**FIGURE 2:**
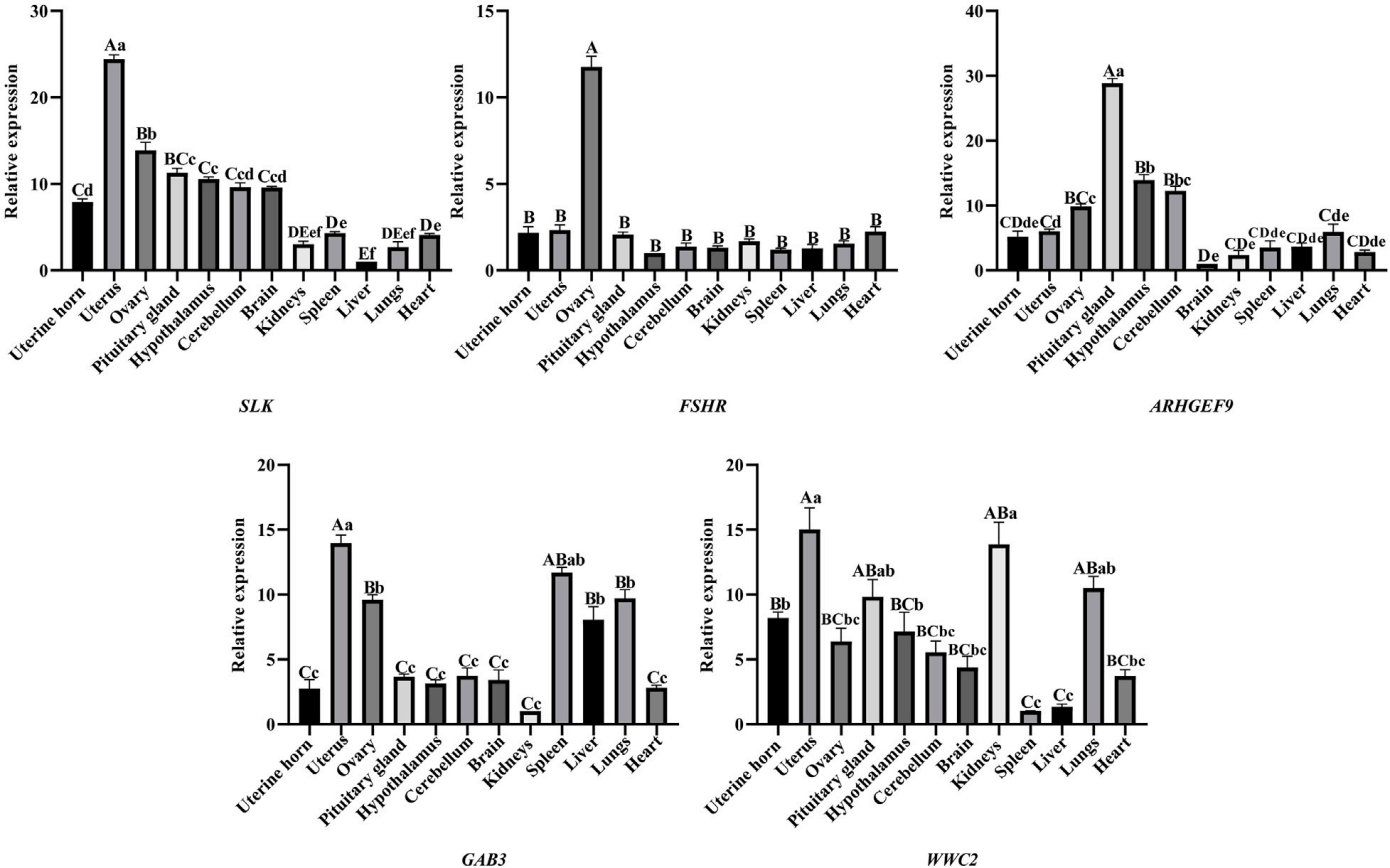
*SLK*, *FSHR*, *ARHGEF9*, *GAB3*, *WWC2* gene expression in different organizations, different capital letters indicate highly significant between groups (*p* < 0.01), different lowercase letters indicate significantly different between groups (*p* < 0.05), and the same letters indicate non-significant different between groups (*p* > 0.05).

The expression distribution pattern of five genes (*WWC2*, *SLK*, *ARHGEF9*, *GAB3*, and *FSHR*) in 12 tissue samples (heart, liver, spleen, lung, kidney, uterine horn, uterus, ovary, hypothalamus, pituitary gland, brain, cerebellum) of sheep was examined by RT-qPCR. The RT-qPCR results are shown in Figure 2. Five genes (*WWC2*, *SLK*, *ARHGEF9*, *GAB3*, and *FSHR*) were expressed in all parts of the sheep’s tissues. The expression of the *SLK* gene in the uterus, the *FSHR* gene in the ovary, and the *ARHGEF9* gene in pituitary and hypothalamus tissues were significantly higher than other parts of sheep’s tissues. The RT-qPCR results showed that *SLK*, *FSHR*, and *ARHGEF9* genes were mainly explicitly expressed in reproductive organs such as the uterus and ovary and tissues related to the hypothalamic-pituitary-gonadal axis and were involved in reproductive effects.

## 4 Discussion

### 4.1 Association of *WWC2* gene polymorphisms with reproductive performance

Previous studies have shown that the *WWC2* gene is strongly associated with reproductive performance. Hermann et al. (2021) found that *WWC2* gene deletion resulted in early embryonic death in mice, and *WWC2* knockout (KO) embryos exhibited growth retardation, disrupted placental development, impaired vascularization, and finally embryonic death, demonstrating that the *WWC2* gene has a significant impact on cell fate determination, cell metabolism, and angiogenesis. In the present study, the relationship between genotype and litter size showed that wild-type CC genotypes in the g.14962207 C>T locus of the *WWC2* gene were significantly correlated with litter size. The litter size of individuals with heterozygous and mutant-pure genotypes was significantly reduced, suggesting that mutations at this locus reduce litter size to a certain extent in the Duolang and Suffolk Sheep. Dysregulated expression of the *WWC2* gene has also been found to be particularly associated with cell-autonomous defects in embryonic cell number and abnormal cell division, typified by an imbalance in chromatin segregation of daughter cells, and during meiotic maturation of mouse oocytes, dysregulation of *WWC2* impedes progression of the fertilizability stage of meiosis II mid-stage arrest, leading to spindle defects and Aurora-A kinase (AURKA) activation failure (Virnicchi et al., 2020).

### 4.2 Association of *SLK* gene polymorphisms with reproductive performance

Ste20 kinase is an important signaling molecule intensively studied in yeast as a regulator of the mitogen-activated protein kinase (MAPK)-dependent pathway in controlling the mating response (Zhao et al., 1995). We obtained the *SLK* gene through previous screening. We found that its expression in reproductive organs such as the uterus and ovary was significantly higher than in visceral tissues such as the heart, liver, and spleen. It was hypothesized that this gene was closely related to reproductive performance in sheep. Studies have shown that the *SLK* gene is commonly expressed in adult tissues, cell lines, and developing mouse embryos (Sabourin and Rudnicki, 1999; Zhang et al., 2002). Song et al. (2023) found that *SLK* is highly expressed in mouse oocyte meiosis with upregulated phosphorylation modifications. *SLK* has also been shown to be required for cell cycle progression from G2 to mitosis (G2/M) by activating the polo-like kinase homolog (PLK1), an essential kinase that regulates cell cycle trafficking, and cell cycle protein-dependent kinases, and that *SLK* depletion results in the inability to downregulate cell cycle protein A and early G2 arrest (Ellinger-Ziegelbauer et al., 2000; O'Reilly et al., 2005). Pryce et al. (2017) found that *SLK* plays a critical role in embryonic development and that knockdown of the *SLK* gene results in embryonic lethality. Consistent with this, the *SLK* gene trap allele (*SLK-LacZ*) that results in the truncation of the ATH structural domain *SLK* was lethal to purebred mouse embryos (Al-Zahrani et al., 2014).

### 4.3 Association of *ARHGEF9* gene polymorphisms with reproductive performance

Based on the association analysis results, we found a significant association between the *ARHGEF9* gene and sheep’s reproductive performance. We hypothesized that this gene may be one of the critical genes affecting sheep’s reproductive performance. The protein encoded by the *ARHGEF9* (guanine nucleotide exchange factor 9) gene is a Rho-like GTPase that switches between an active (GTP-bound) state and an inactive (GDP-bound) state that is important for energy metabolism (Ibaraki et al., 2018). Previous studies have shown that the *ARHGEF9* gene is associated with critical genes *DIO2*, *DIO3*, *TSHB*, and *TSHR* in the thyroid (TH) axis pathway, which may be related to the promotion of estrus (Malpaux et al., 2001; Fan et al., 2022). The thyroid (TH) axis is essential for seasonal reproduction in sheep, and thyrotropin secreted by the nodal section (PT) is the major photoperiod-dependent upstream regulator of (*DIO2*/*DIO3*) gene expression. Long sunlight promotes thyrotropin production, increasing type II thyroid hormone deiodinase (*DIO2*) and inhibiting *DIO3* expression, which enhances TH signaling in the MBH. In contrast, short sunlight has the opposite effect. Overall, the TH axis directs seasonal reproduction through thyroid hormone (TH) reciprocal regulation of TH deiodinase (*DIO2*/*DIO3*) gene expression in stretch cells in the medial ventricular zone (MBH) of the hypothalamus (Goldman, 2001; Hanon et al., 2008). Our study selected seasonal rutting sheep (Duolang and Suffolk Sheep) and year-round rutting sheep (Hu Sheep) as test subjects. We used the KASP technique to type the mutant locus of the *ARHGEF9* gene and then found differences in the typing results of the gene in the three populations. Maybe due to breed or geographical differences, only the wild-type CC genotype was typed in the Suffolk Sheep populations. In the Duolang Sheep populations, two genotypes (wild-type CC and heterozygous CA) were typed. Three genotypes (wild-type CC, heterozygous CA, and mutant-pure AA genotypes) were typed in the Hu Sheep populations. The *ARHGEF9* gene did not appear in the mutant-pure individual in the seasonally rutting sheep population. In contrast, the mutant-pure AA genotype individual appeared in the year-round rutting Hu Sheep populations, and we hypothesized that it might be due to this pure mutation that causes year-round estrus in the Hu Sheep populations. Currently, there are fewer studies on the role of the *ARHGEF9* gene in sheep reproductive performance. The regulatory mechanism is unclear and its function needs to be further verified.

## 5 Conclusion

Breeding high fecundity sheep is very important for sheep meat production and molecular marker screening can be used to improve early molecular selection, to make breakthroughs in the development of genes and markers for multiple births in sheep, to improve the efficiency of molecular breeding for high fecundity sheep, and also to improve other low yielding populations to increase the efficiency of meat production in sheep. The present study verified the role of five genes. Among the five selected genes, the *WWC2* gene g.14962207 C>T locus, the *SLK* gene g.27108855 G>A locus, the *ARHGEF9* gene g.48271079 C>A locus, and the *FSHR* gene g.80789180 T>G locus were identified as having significant molecular markers among different genotypes. These four loci can be used to detect the local sheep populations in Xinjiang (Duolang Sheep) and introduced sheep populations (Suffolk and Hu Sheep) in terms of litter size. This finding provides new information to explain sheep fertility’s regulatory mechanisms and identify molecular markers for litter size traits. These molecular markers are essential for the selection of high fecundity sheep breeds.

## Data Availability

The original contributions presented in the study are included in the article/Supplementary Material, further inquiries can be directed to the corresponding authors.
